# Report of Committee on Alloys

**Published:** 1908-09-15

**Authors:** G. S. Allen, A. H. Merritt, J. Morgan Howe


### REPORT OF COMMITTEE ON ALLOYS.

BY DRS. G. S. ALLEN, A. H. MERRITT AND J. MORGAN HOWE.

Your committee on alloys respectfully reports as follows: Analyses of six of the popular proprietary alloys used for dental amalgams have been obtained from Dr. P. S.

Burns, of the Massachusetts Institute of Technology, whose report is herewith presented.

Analyses of alloys for The New York Institute of Stomatology:

| | % Silver | % Tin | % Copper | % Zinc | % Gold |
|---|---|---|---|---|---|
| 1. Twentieth Century | 67.03 | 27.13 | 4.87 | 1.10 | ---- |
| 2. True Dentalloy | 65.91 | 27.13 | 5.21 | 1.52 | ---- |
| 3. Standard Alloy | 53.55 | 35.03 | 2.76 | ---- | 8.82 |
| 4. Fellowship Alloy | 67.45 | 26.80 | 5.73 | 0.55 | ---- |
| 5. Odontographic Alloy | 66.87 | 26.48 | 6.21 | trace | 0.28 |
| 6. C. Ash. Sons Co. (C. A. S.) Alloy | 66.54 | 27.16 | 5.02 | 0.90 | ---- |

We would call attention to the fact that the composition of four of these alloys is practically the same. The slight variation in the proportions of their constituents may make a perceptible difference in their working and setting qualities, but the cost of material for their manufacture is practically the same. Yet some of these secret formula products are sold at double the price of others.

We think it safe to assume that the practical identity of these compounds has been known to each of their manufacturers since their introduction, and we regard it a lesson worthy of consideration, that the makers of the highest priced compounds have enjoyed the benefits of such extra profit without any revelation of the similarity of composition being made by those making practically the same alloy for one-half the price. It would seem that the maintenance of that complaisant acceptance of secret formula products which has generally characterized the dental body, is so valuable an asset to the purveyors of supplies that they make common cause in "standing pat" on this point, notwithstanding the competition that exists to market their products.

Another of these alloys has, as reported, a composition almost identical with the average of the four mentioned above, excepting that about one-quarter of a part of gold in one hundred parts of alloy, is substituted for the small constituent of zinc in the other similar alloys.

The composition of the last alloy to which we have our attention directed by these analyses is decidedly different from the other five in the proportion of constituents, containing less silver and less copper, more tin, and nearly nine parts of gold to the hundred parts of the compound. This alloy we believe has been upon the market many years, long before Dr. Black published the reports of his studies of this subject. But it is worthy of note in regard to these alloys whose analyses report such marked similarity that for each one of them we think it has been claimed that they are "made after Dr. Black's method." The method, we understand, refers to manipulation in the process of making, and Dr. Black is on record over his signature, on a circular relating to one of these alloys, stating that, "Any one who knows the methods which I have developed, and has made a sufficient study of the metals to enable him to make alloys understandingly, needs no formulae."—*The Journal.*

